# Synergy of Taxol and rhein lysinate associated with the downregulation of ERK activation in lung carcinoma cells

**DOI:** 10.3892/ol.2013.1398

**Published:** 2013-06-14

**Authors:** YONG-ZHAN ZHEN, GANG HU, YU-FANG ZHAO, FENG YAN, RAN LI, JUN-LING GAO, YA-JUN LIN

**Affiliations:** 1 Basic Medical College of Hebei United University, Tangshan, Hebei 063000, P.R. China; 2 The Key Laboratory of Geriatrics, Beijing Hospital & Beijing Institute of Geriatrics, Ministry of Health, Beijing 100730, P.R. China

**Keywords:** rhein lysate, Taxol, lung cancer, combination therapy, extracellular signal-regulated kinase

## Abstract

In previous studies we observed that rhein lysinate (RHL), a salt of rhein and lysine that is easily dissolved in water, inhibited the growth of tumor cells in breast cancer, ovarian cancer, hepatocellular carcinoma and cervical cancer. The present study aimed to investigate the effects of RHL on H460 and A549 non-small cell lung cancer (NSCLC) cells using a combination of RHL and Taxol. A 3-(4,5-dimethylthiazol-2-yl)-2,5-diphenyl-tetrazolium bromide (MTT) assay was used to determine the growth inhibition effect of the drugs in the H460 and A549 cells. Cell apoptosis was analyzed by flow cytometry combined with fluorescein-isothiocyanate-Annexin V/propidium iodide (PI) staining. The expression levels of proteins were detected by western blotting. There was a significant reduction in the proliferation of the NSCLC cell lines treated with a combination of Taxol and RHL. The overall growth inhibition was directly correlated with apoptotic cell death. RHL potentiated Taxol-induced cell killing by reducing extracellular signal-regulated kinase (ERK) activity and increasing the levels of cleaved poly(ADP-ribose) polymerase (PARP) and caspase-3. Notably, the results for the Bcl-2 and NF-κB proteins also showed downregulation in the combined treatment group compared with the single-agent treatment and the untreated control groups. The present results showed that RHL potentiates the growth inhibition induced by Taxol in NSCLC cells and showed that this synergy may be associated with the downregulation of ERK activation.

## Introduction

Lung cancer is one of the most harmful malignant tumors to human health and life and its incidence is increasing every year, ranking first in large- and medium-sized cities in the incidence of malignant tumors (1,2). Two-thirds of patients are in the advanced stage when diagnosed with lung cancer and lose the opportunity for surgical treatment. Non-small cell lung cancer (NSCLC) comprises 80% of all lung cancers. Chemotherapy is primarily used for the treatment of advanced lung cancer. Although new anticancer drugs and chemotherapies have been introduced, the outcomes for certain patients are not always satisfactory and patients become less able to tolerate treatment as the chemotherapy extends (3).

Therefore, targeted therapy for lung cancer has become a research hotspot in recent years. Erlotinib and gefitinib are widely used in lung cancer therapy, although they are only effective for specific pathological situations and patients; the outcomes for the rest of the patients remain unsatisfactory. The issue of subsequent resistance to chemotherapy and the mechanisms involved have yet to be elucidated. Novel treatment strategies targeting this aggressive disease are expected to offer long-term disease control or possibly even a cure.

Rhein, one of the major bioactive constituents of the rhizome of rhubarb (4,5), inhibits the proliferation of various human cancer cells (6–10). Our previous studies showed that rhein lysinate (RHL), a salt of rhein and lysine that is easily dissolved in water, exhibits anticancer activity in breast cancer, ovarian cancer, hepatocellular carcinoma and cervical cancer *in vivo* and *in vitro* (11–14).

We previously showed that RHL was highly active in targeting the MEK/extracellular signal-regulated kinase (ERK) signal pathway and induced apoptosis and cell cycle arrest in human ovarian cancer cells (12). The present study aimed to determine whether RHL has additive or synergistic effects with Taxol and to determine the molecular mechanisms by which RHL enhances Taxol-induced cytotoxicity and apoptosis.

## Materials and methods

### Chemicals and reagents

Rhein (purity, 98%) was purchased from Nanjing Qingze Medicine Ltd., (Nanjing, Jiangsu, China), while lysine was purchased from Beijing Solarbio Science and Technology Co. (Beijing, China). RHL was made in our department (patent no. 200810089025.8). The 3-(4,5-dimethylthiazol-2-yl)-2,5-diphenyl-tetrazolium bromide (MTT) and dimethyl sulfoxide (DMSO) were obtained from Sigma Aldrich (Shanghai, China). Antibodies targeting poly(ADP-ribose) polymerase (PARP), caspase-3, Bcl-2, NF-κB, MEK, p-MEK, ERK, p-ERK and β-actin were purchased from Santa Cruz Biotechnology, Inc. (Santa Cruz, CA, USA). Secondary antibodies against rabbit or mouse IgG were purchased from Cell Signaling Technology (Danvers, MA, USA). The prestained protein marker p7708V was purchased from New England Biolabs Ltd. (Beijing, China). All other chemicals were of standard analytical grade.

### Cell culture

The human lung carcinoma cell lines, H460 and A549, were obtained from the Cell Center of the Institute of Basic Medical Sciences, Chinese Academy of Medical Sciences and Peking Union Medical College (Beijing, China). The cells were cultured in RPMI 1640 (Gibco BRL, Grand Island, NY, USA) supplemented with 10% heat-inactivated fetal bovine serum (Sigma Chemical Co., St. Louis, MO, USA), 2 mM glutamine, 100 U/ml penicillin and 100 *μ*g/ml streptomycin at 37°C in a humidified atmosphere containing 5% CO_2_.

### Cell proliferation assay

Cell proliferation assays were performed using the MTT method, according to the manufacturer’s instructions. The cells were seeded in 96-well plates (Costar, Cambridge, MA, USA) with 2,500 cells/well. Subsequent to overnight incubation, triplicate wells were treated with various concentrations of RHL for 48 h. Next, 20 *μ*l MTT solutions (5 mg/ml in PBS) were added to each well and incubated for 4 h at 37°C. The MTT formazan was dissolved in 150 *μ*l DMSO and the absorbance was measured with a microplate reader (Multiskan MK3; Thermo Labsystem, Waltham, MA USA) at a wavelength of 570 nm.

### FITC-Annexin V/PI apoptosis assay

The cells were collected and resuspended in 200 *μ*l binding buffer, then 10 *μ*l FITC-labeled enhanced Annexin V (Baosai Biotechnology Ltd., Beijing, China) and 100 ng propidium iodide (PI) were added. Upon incubation in the dark for 15 min at room temperature or 30 min at 4°C, the samples were diluted with 300 *μ*l binding buffer. Flow cytometry was performed on a FACScan instrument (Becton-Dickinson, Franklin Lakes, NJ, USA) and the data were processed using WinMDI/PC-software.

### Western blot analysis

The cells were harvested and washed with PBS solution. The whole cellular extracts were prepared by incubating the cells on ice in lysis buffer containing 50 mM Tris-HCl (pH 7.5), 150 mM NaCl, 2 mM EDTA, 2 mM EGTA, 1 mM dithiothreitol, 1% Nonidet P-40, 0.1% SDS, protease inhibitors (1 mMPMSF, 5 *μ*g/ml aprotinin, 5 *μ*g/ml leupeptin and 5 *μ*g/ml pepstatin) and phosphatase inhibitors (20 mM β-glycerophosphate, 50 mM NaF and 1 mM Na_3_VO_4_). The supernatant was collected through centrifugation at 12,000 x g for 12 min. Protein concentrations were determined with the Bradford protein assay. Equal amounts of lysate (40 *μ*g) were resolved by SDS-PAGE and transferred to polyvinylidene difluoride membrane (Millipore Corp., Bedford, MA, USA). The membranes were blocked in TBST containing 5% skim med milk at room temperature for 2 h and probed with primary antibodies overnight at 4°C. The membranes were then blotted with an appropriate horseradish peroxidase-linked secondary antibody (Santa Cruz Biotechnology, Inc.). Proteins were visualized using enhanced chemiluminescence western blotting detection reagents (Amersham Pharmacia Biotech, Inc., Piscataway, NJ, USA).

## Results

### Taxol-induced growth inhibition is potentiated by RHL in H460 and A549 cells

**Figure 1.**
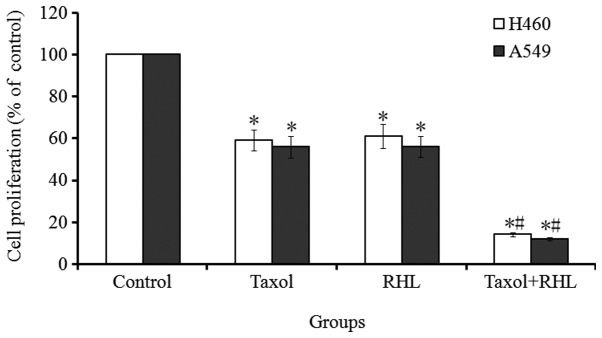
Growth inhibition of H460 and A549 cells treated with Taxol (1 *μ*M), RHL (100 *μ*M) and a combination of the two, evaluated with MTT assays. There was a significant reduction in cell growth in the two cell lines treated with Taxol and RHL compared with the cells treated with taxol alone. ^*^P<0.05 vs. control group. ^#^P<0.05 vs. Taxol or RHL alone groups. RHL, rhein lysinate.

The growth of the H460 and A549 cells treated with RHL (100 *μ*mol/l), Taxol (1 *μ*mol/l) or a combination of the two was determined via MTT assays. The dose used in the present study was selected based upon a preliminary dose escalation study. A significant reduction in growth was observed in the cells treated with RHL and Taxol in combination compared with treatment with RHL or Taxol alone (Fig. 1). The combined drug intoxication (CDI) value was <0.7, indicating that the two drugs have a synergistic effect.

### Taxol-induced apoptosis is sensitized by RHL in H460 and A549 cells

**Figure 2.**
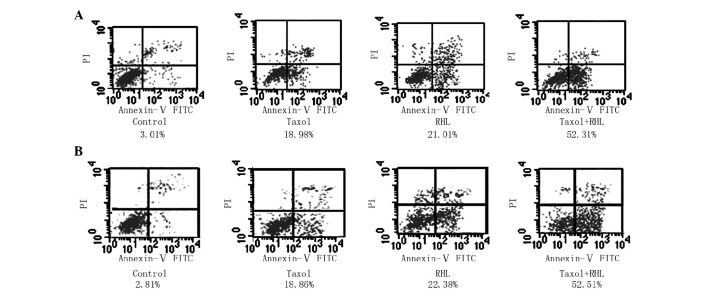
Induction of apoptosis in (A) H460 and (B) A549 cells treated with Taxol (1 *μ*M), RHL (100 *μ*M) and a combination of the two, as evaluated by FITC-Annexin V/propidium iodide (PI) apoptosis assays. A significant potentiation of apoptosis was observed in the cells treated with a combination of Taxol and RHL compared with the cells treated with Taxol alone. RHL, rhein lysinate.

The induction of apoptosis was observed in the lung cancer cells treated with either Taxol, RHL or a combination of the two. Relative to the single agents, the combined treatment induced more apoptosis in the two cell lines, as shown by flow cytometry combined with FITC-Annexin V/PI staining (Fig. 2). The ratios of apoptosis were 52.31 and 52.51% in the combined treatment groups, whereas the ratios of the Taxol groups were 18.98 and 18.86% in the H460 and A549 cells, respectively. These results were consistent with the cell growth inhibition experiments using MTT, suggesting that the loss of viable cells due to RHL and Taxol treatment is partly due to the induction of an apoptotic cell death mechanism.

### Taxol-induced apoptosis signaling is augmented by RHL in H460 and A549 cells

**Figure 3.**
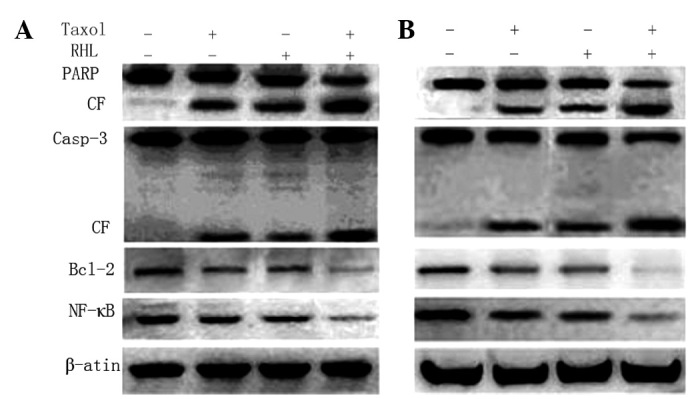
H460 and A549 cells were treated with Taxol (1 *μ*M), RHL (100 *μ*M) and the two drugs in combination for 48 h. The levels of caspase-3, PARP, Bcl-2, NF-κB and β-actin in the (A) H460 and (B) A549 cells were determined by western blotting analysis. RHL, rhein lysinate; PARP, poly(ADP-ribose) polymerase.

In an attempt to investigate the mechanism of the enhanced apoptotic process induced by the treatment of the cells with RHL and Taxol, the levels of caspase-3 and PARP were assessed in the H460 and A549 cells. The results showed that the combined treatment was able to increase the levels of cleaved PARP and caspase-3 significantly. The results for the anti-apoptotic Bcl-2 and NF-κB proteins also showed downregulation in the combination group relative to the single-agent treatments and untreated control (Fig. 3).

### Effects of Taxol, RHL and a combination of the two on the MEK/ERK signaling pathway

**Figure 4.**
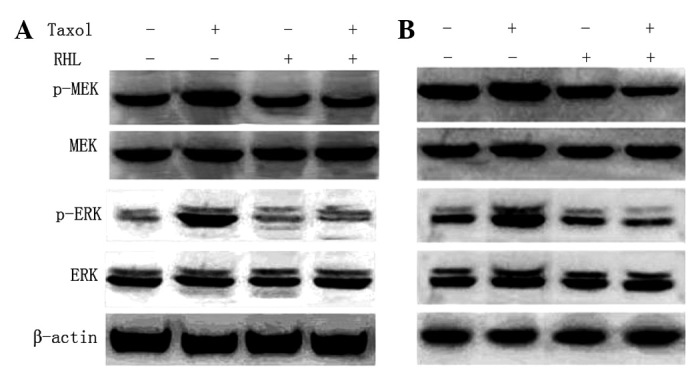
Levels of MEK, ERK and β-actin in (A) H460 and (B) A549 cells treated with Taxol (1 *μ*M), RHL (100 *μ*M) and a combination of the two for 48 h were determined by western blotting analysis. ERK, extracellular signal-regulated kinase; RHL, rhein lysinate.

In the present study, it was observed that Taxol treatment enhanced the activation of ERK in the H460 and A5449 cells. However, Taxol in combination with RHL prevented the Taxol-induced ERK activation through the inhibition of MEK phosphorylation (Fig. 4).

## Discussion

Lung cancer remains the leading cause of cancer-related mortality worldwide despite advances in the field of cancer therapeutics (2). Traditional treatment with empirically selected cytotoxic chemotherapeutic agents has provided small, but real survival benefits (2). Moreover, cancer recurrence and subsequent resistance to chemotherapy remain problematic and the mechanisms are not clear. New agents are required to offer long-term disease control or even possibly a cure. Advances and insights into the molecular pathogenesis of lung cancers have provided certain novel molecular targets, offering new strategies and agents that are tumor specific.

Certain studies have demonstrated that tumor cells are able to produce resistance to Taxol by activating the MEK/ERK signal pathway. The activation of ERK was demonstrated to be important in mediating proliferation in cancer cells (15–18). Inhibiting MEK/ERK signaling may therefore enhance Taxol-induced cytotoxicity in lung cancer cells.

Rhein, one of the major bioactive constituents of the rhizome of rhubarb (4,5), inhibits the proliferation of various human cancer cells (6–10). We previously demonstrated that RHL, a salt of rhein and lysine that is easily dissolved in water, has anticancer activity in breast cancer, ovarian cancer, cervical cancer and hepatocellular carcinoma *in vivo* and *in vitro* (11–14). We also showed that RHL was highly active in targeting the MEK/ERK signal pathway and that it induced apoptosis and cell cycle arrest in human ovarian cancer cells (12).

In the present study, it was observed that RHL improved the anti-tumor activity of Taxol in lung cancer. Mechanically, RHL potentiated Taxol-induced cell killing by reducing the phosphorylation of ERK and increasing the levels of cleaved caspase-3 and PARP.

These caspases belong to a family of cysteine proteases whose activation induces cellular apoptosis. Specifically, proteolytically cleaved caspase-3 and caspase-7, the active forms of pro-caspase-3 and pro-caspase-7, are key molecules for identifying the activation of apoptosis (19–21). In addition, PARP is one of the main substrates of activated caspase pathways and a well-established indicator of apoptotic cell death.

The results for the anti-apoptotic Bcl-2 and NF-κB proteins also showed downregulation in the combined treatment group compared with the signal-agent treatment and untreated control groups.

It is known that members of the Bcl-2 protein family act as key regulators of cellular apoptosis and are important determinants of cellular sensitivity or resistance to chemotherapy drugs (22–24). The overexpression of Bcl-2, an anti-apoptosis member of this family, is commonly observed in human lung cancer and Bcl-2 overexpression correlates with chemoresistance in this disease. In addition, NF-κB has been shown to inhibit apoptosis in response to chemotherapeutic agents. Compounds targeting the NF-κB pathway are able to sensitize lung tumor cells by counteracting resistance mechanisms, and therefore, deserve further evaluation with regard to chemotherapy and the possible chemoprevention of lung cancer (25–27).

In conclusion, the present findings showed a synergistic effect between RHL and Taxol in certain lung cancer cell lines. This synergy is likely to be associated with the downregulation of ERK activation. Accordingly, further mechanistic studies may be useful in the treatment of patients with lung carcinoma.
